# Supplementary figures and images for: Variable Expression of GABAA Receptor Subunit Gamma 2 Mutation in a Nuclear Family Displaying Developmental and Encephalopathic Phenotype
Source: Int J Mol Sci. 2022 Aug 26;23(17):9683. doi: 10.3390/ijms23179683 (PMC9456057; doi:10.3390/ijms23179683)

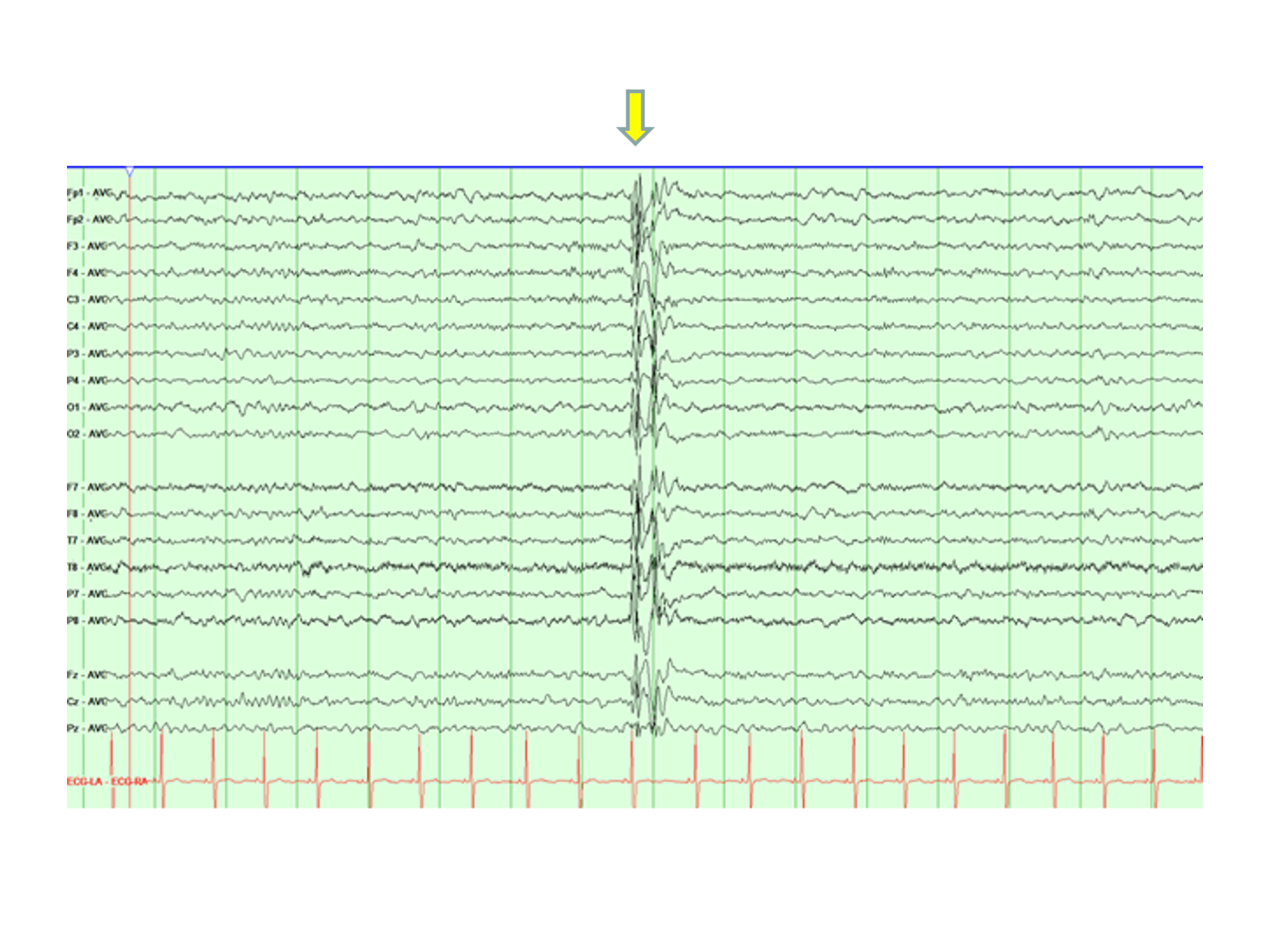

Supplement: Supplementary file 1 [file ijms-23-09683-s001.zip › ijms-1852738-supplementary/Supplemental Files/Figure S1.tiff]

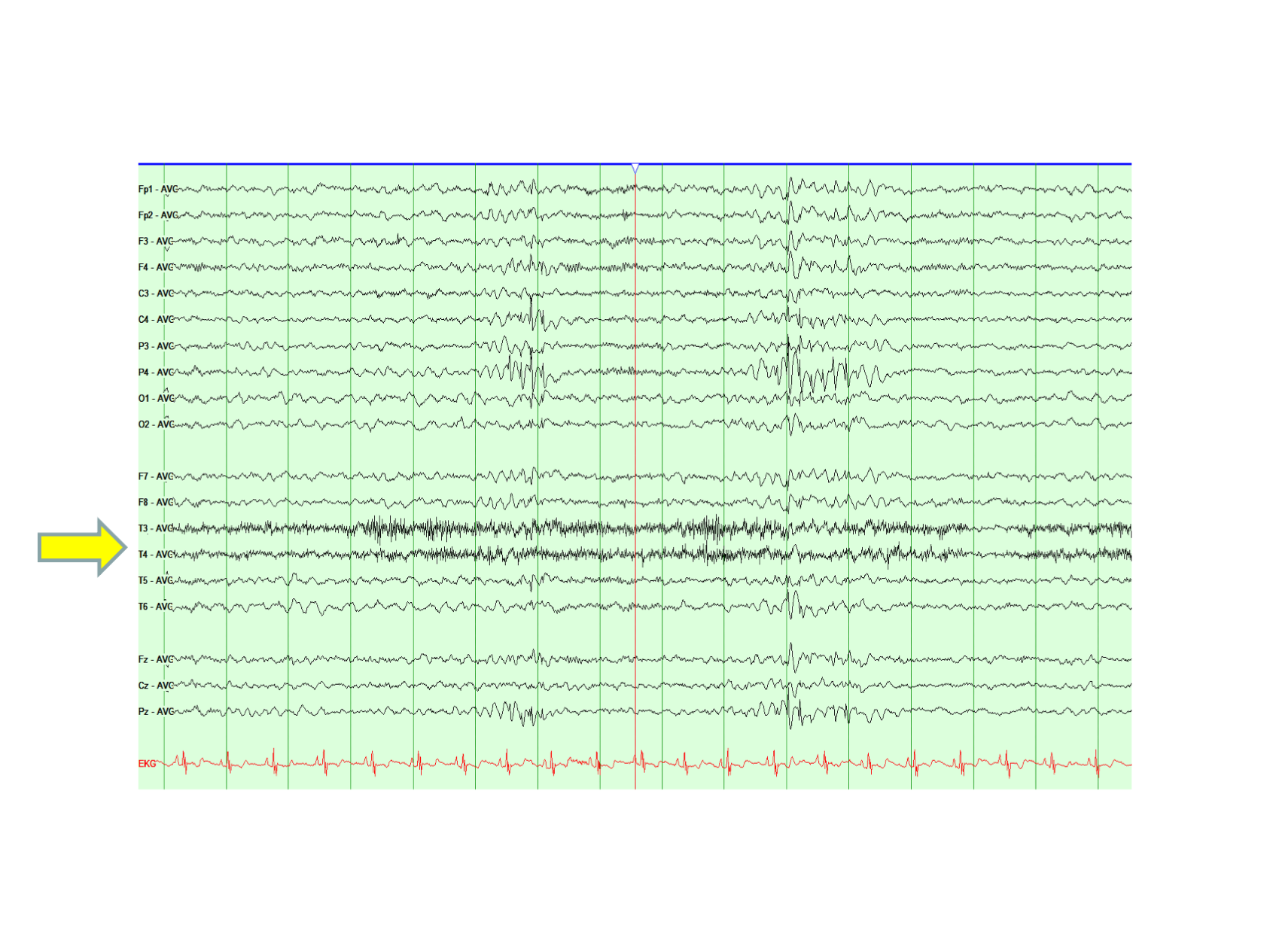

Supplement: Supplementary file 1 [file ijms-23-09683-s001.zip › ijms-1852738-supplementary/Supplemental Files/Figure S2.tiff]

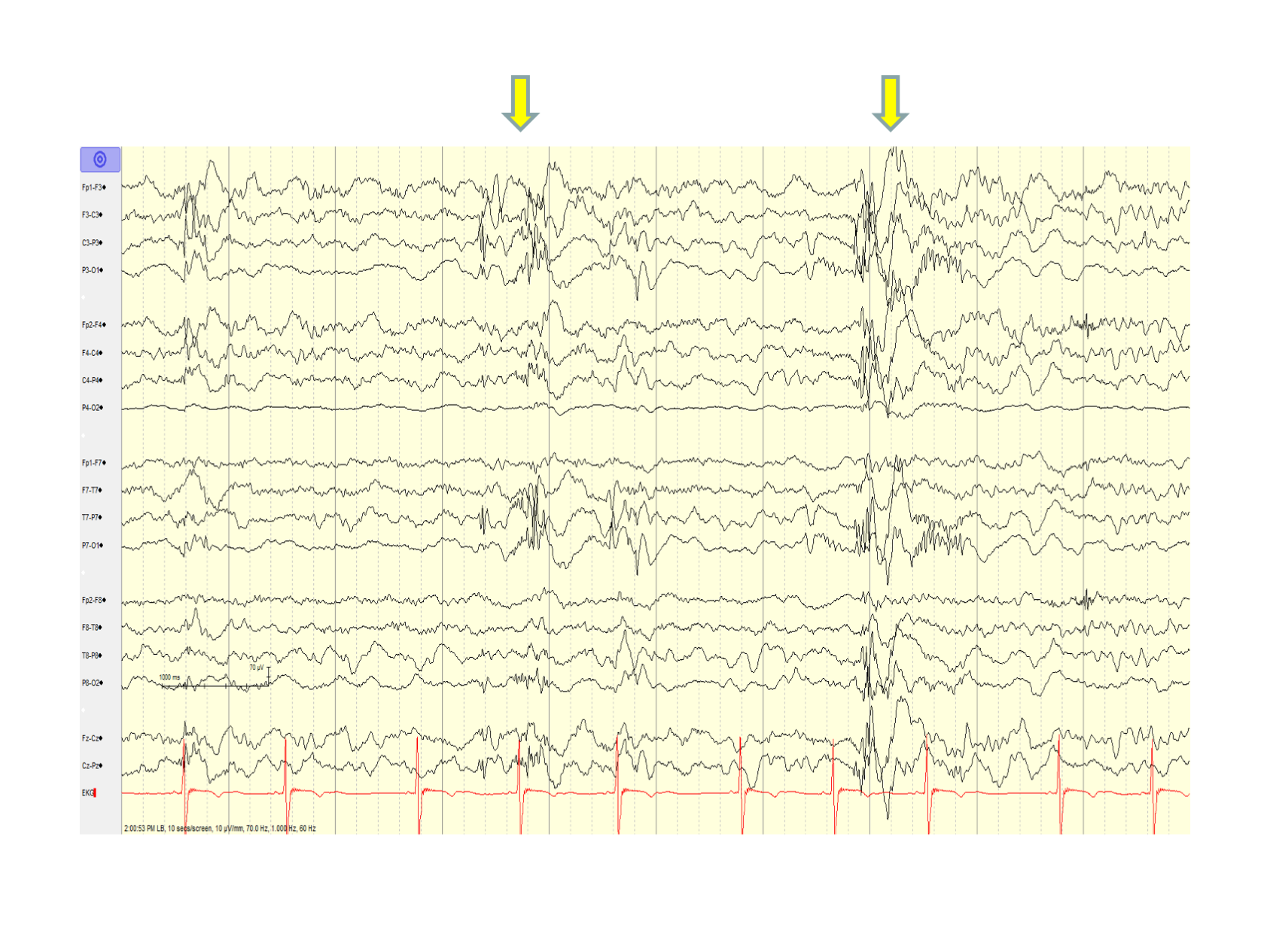

Supplement: Supplementary file 1 [file ijms-23-09683-s001.zip › ijms-1852738-supplementary/Supplemental Files/Figure S3.tiff]
